# Association between Urinary Levels of Aflatoxin and Consumption of Food Linked to Maize or Cow Milk or Dairy Products

**DOI:** 10.3390/ijerph17072510

**Published:** 2020-04-06

**Authors:** Fulvio Ferri, Carlo Brera, Barbara De Santis, Giorgia Collini, Enrica Crespi, Francesca Debegnach, Angelo Gargano, Daniela Gattei, Ines Magnani, Pamela Mancuso, Stefania Mozzanica, Elvira Teodori, Olivera Djuric, Paolo Giorgi Rossi

**Affiliations:** 1 Servizio Prevenzione Sicurezza Ambienti di Lavoro (SPSAL), Azienda Unità Sanitaria Locale—Reggio Emilia—IRCCS, Via Amendola 2, 42122 Reggio Emilia, Italyenrica.crespi@ausl.re.it (E.C.); angelo.gargano@ausl.re.it (A.G.); daniela.gattei@ausl.re.it (D.G.); magnanines@gmail.com (I.M.); stefania.mozzanica@ausl.re.it (S.M.); 2 Laboratorio Nazionale di Riferimento (LNR) per le Micotossine—Istituto Superiore di Sanità, Roma, Viale Regina Elena 299, 00161 Rome, Italy; carlo.brera@iss.it (C.B.); barbara.desantis@iss.it (B.D.S.); francesca.debegnach@iss.it (F.D.); 3 Servizio di Epidemiologia, Azienda Unità Sanitaria Locale—IRCCS di Reggio Emilia, Via Amendola 2, 42122 Reggio Emilia, Italy; pamela.mancuso@ausl.re.it (P.M.); olivera.djuric@ausl.re.it (O.D.); paolo.giorgirossi@ausl.re.it (P.G.R.); 4 Laboratorio Analisi, Azienda Unità Sanitaria Locale—IRCCS di Reggio Emilia, Via Amendola 2, 42122 Reggio Emilia, Italy; elvira.teodori@ausl.re.it; 5 Center for Environmental, Nutritional and Genetic Epidemiology (CREAGEN), Section of Public Health, Department of Biomedical, Metabolic and Neural Sciences, University of Modena and Reggio Emilia, Via Università 4, 41121 Modena, Italy

**Keywords:** aflatoxin M1, aflatoxins, animal feed, maize, occupational exposure

## Abstract

The aim of this analysis was to assess the association between consumption of maize and dairy products and urine and serum levels of aflatoxin FM1 (AFM1) in a sample of 59 males occupationally exposed (29) and non-exposed (30) to aflatoxins. Two urine samples were collected for each person; each sample was accompanied by a questionnaire on food consumption in the preceding 96 h. Given the similar levels of contamination found in exposed and non-exposed workers, the association between food consumption and AFM1 levels was analyzed by pooling samples from exposed and non-exposed workers. No serum sample was found to be positive for AFM1, whereas 74% of the urine samples were positive; the average concentration of positive samples was 0.042 ng/mL (range < limit of detection (LoD) (0.002)–0.399 ng/mL). Of the 21 samples from maize consumers, 13 were positive for AFM1 (62%), with a mean concentration of 0.026 ng/mL (range 0.006–0.088 ng/mL), while 76% (74/94) of the samples from maize non-consumers were positive (mean 0.045, range < LoD (0.002)–0.399 ng/mL). No association was found with milk or dairy products. The high urine level of aflatoxins found in both exposed and non-exposed workers was not associated with the consumption of maize or cow milk products.

## 1. Introduction

Aflatoxins, produced by two species of *Aspergillus* (*A. flavus* and *A. parasiticus*), a mold growing on plants in warm and humid climates [1], are carcinogenic to humans. In fact, the International Agency for Research on Cancer classifies aflatoxins as carcinogenic to humans (group 1), with sufficient evidence for such carcinogenic agents as aflatoxins B1, G1, and M1 [2].

Humans are primarily exposed to aflatoxins through food ingestion [3]; the foods most often contaminated are peanuts, other nuts with shells, maize, rice, cereals, dried fruit, spices, and oils derived from vegetable seeds. Since many of these seeds are also used to feed animals, secondary contamination is also possible through milk and dairy products; there is no accumulation of aflatoxins in meat, as their catabolism is quite fast [4,5].

In 2006, the European Commission introduced measures to reduce the risk of consumer exposure by defining procedures to sample and check foods that are known to be at risk of aflatoxin contamination and by setting thresholds for the presence of aflatoxins for commercialized products and foodstuffs [6]. Nevertheless, food contamination still occurs, and contaminated foods reach consumers.

The short-term exposure to aflatoxins in humans can be monitored through urine and serum levels of toxin catabolites [7,8,9,10,11].

In 2012, the particularly dry summer favored contamination of maize in the Po Valley of Northern Italy [12,13]. In this area, maize is mostly used to feed cows for cheese production. A strict control campaign by the public health authorities led to identifying and stocking an enormous quantity of maize that was not good for foodstuffs. The accumulation of this contaminated maize carried two main risks. The first was that workers in the several mills producing foodstuffs that developed decontamination procedures might be exposed to dust. The second was that dairy products might be contaminated, since some contaminated foodstuffs might have eluded control measures.

In the spring of 2013 and 2014, the Reggio Emilia local health authority conducted a study to assess the risk of occupational exposure to aflatoxins at the two mills processing contaminated maize [14]. To conduct this study, a questionnaire on food consumption in the 96 h prior to urine and serum sample collection was administered both to exposed and non-exposed workers; urine and serum samples were then collected and tested for aflatoxin. The study found that urine samples of exposed and non-exposed workers had similar proportions of positivity for AFM1, 71% and 77%, respectively, with an average concentration of 0.035 and 0.027 ng/mL in exposed and non-exposed workers, respectively. These values are very high when compared to other European studies: Gerding and coll. and Heyndrickx and coll. found no contaminated samples and had similar analytical accuracy with the limit of detection (LoD) ranging from 0.0013 to 0.0025 ng/mL [15,16]; Solfrizzo reports a limit of quantification (LoQ) of 0.02 ng/mL, and only 6% of samples over the LoQ [17]; in the Reggio Emilia study [14], 33% of the samples were over 0.02 ng/mL. Only surveys from industrialized countries with a tropical climate have found similar proportions of contaminated samples, although concentration values of aflatoxins were about ten times lower than those observed in our study [8,18,19,20,21,22]; similar levels of contamination have been observed only in the surveys conducted in Africa and Asia [23,24,25,26,27,28,29]. Even though some results of the 2017 Reggio Emilia survey might suggest a small contribution from occupational exposure, it was clear that other sources of aflatoxins were present for the general population, with the most plausible exposure being food. Specifically, the hypothesis was that contaminated maize could have entered the food chain, thereby leading to an exposure for humans.

The aim of this paper is to assess the association between consumption of foods in the preceding 96 h that could be a primary or secondary source of maize-related aflatoxin contamination, i.e., maize-containing foods and dairy products, and urine and serum levels of aflatoxins in a population of male workers employed at two mills and at the local health authority.

## 2. Materials and Methods

### 2.1. Setting

The study was conducted under the supervision of the local health authority (LHA) of Reggio Emilia and was approved by the ethics committee of the Reggio Emilia province (act number 2013/DS/0086, 30/12/2013).

Our data come from a survey on food consumption and urine and serum aflatoxin levels designed to study potential occupational exposure at two mills in Northern Italy; that survey involved 30 exposed workers and 30 non-exposed controls [14]. Plant A is a large foodstuff plant that produces flour, compost, and pellet fuel, mostly derived from maize, and Plant B is a small cereal drying and sorting plant that processes mainly maize.

The study was agreed on with the plant management, trade union representatives, and the competent medical team of the companies involved according to the criteria and principles set forth by the Italian legislation on occupational health and safety. The exposed and non-exposed workers were informed about the purpose of the study in the course of a public meeting, after which formal consent for participation was individually requested and signed.

### 2.2. Study Design

The study design and methods were described elsewhere [14]. Briefly, the study included only males, since the exposed workers were mostly males. The 30 exposed workers included in the study population were 3 workers employed at Plant B and 27 workers from Plant A (randomly extracted from the 51 workers who consented to participate who were present on the days of sampling). The non-exposed controls included 9 non-exposed workers of Plant A (administrative employees) and 21 LHA workers randomly extracted from the 152 workers who agreed to participate.

**Figure 1:**
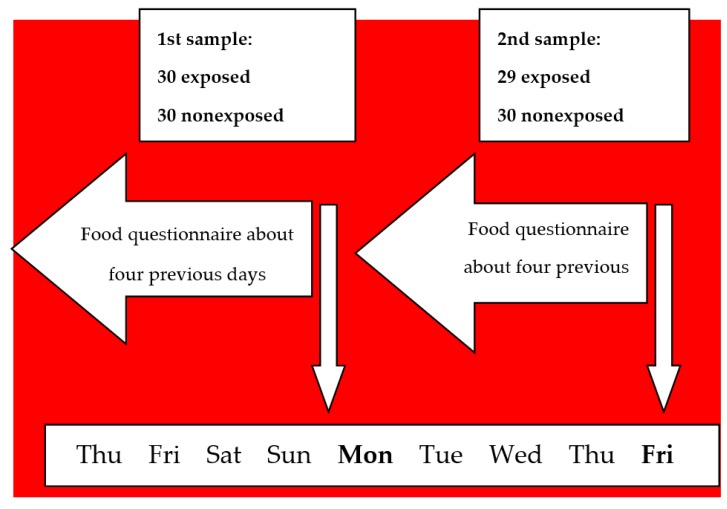
Scheme of the days investigated for food consumption.

Blood and urine samples were collected on the Monday morning to have one sample after about 64 h of washing-out, i.e., from the end of the workday on Friday to Monday morning, and again on the following Friday morning to have one sample at the end of the working week with the maximum exposure to mill dust. Thus, 120 samples were planned: 30 exposed and 30 non-exposed samples collected in both groups on Monday and Friday. The questionnaires collecting the data on food consumption in the preceding four days were administered at the moment blood and urine samples were taken (Figure 1).

### 2.3. Urine and Blood Samples Collection and Analysis

Human biological samples were collected by a physician and a nurse between 31 March 2014 and 18 April 2014, at the two plants.

Serum and urine samples were stored and then dispatched under controlled temperature (−20 °C) to the national reference laboratory (NRL) for mycotoxins in Rome (Istituto Superiore di Sanità).

Analyses were carried out for AFB1, AFB2, AFG1, AFG2, AFM1, and AFOH in the serum and urine samples collected on Monday and Friday from exposed and non-exposed workers.

Detailed methods of extraction and detection of all the searched aflatoxins from urine and serum, as well as the most important validation parameters (relative standard deviation of repeatability, recovery, limit of detection (LoD)), trueness, and overall findings are reported elsewhere [14]. Since none of the serum samples was positive for any of the tested aflatoxins, and AFM1 was by far the most frequently found aflatoxin and the most clinically relevant [2], in the present work, analyses were restricted to AFM1 urinary levels. Given small and non-significant differences between the average aflatoxin levels found in urine samples of the exposed and non-exposed workers, the analyses were conducted on the overall study sample. Stratified analyses by occupational exposure are presented in Table S1.

### 2.4. Food Questionnaire

In order to rule out possible confounding in the association between the urine aflatoxin level and the occupational exposure due to food contamination, the survey included questionnaires on the food consumed in the 4 days before urine and serum sampling. Before taking a blood sample, the questionnaire was administered to all the participants by a nurse. Food products that are a potential source of aflatoxin contamination, such as corn and other cereals, rice, spices, nuts, dried fruits, dehydrated fruits, fresh milk, fresh cheese and hard cheese, other dairy products were the main focus of the questionnaire. In order to mask the risk factors known to the interviewee, some other foods known not to be a risk of aflatoxin exposure (i.e., fish, shellfish, meat, fresh fruit) were also included in the questionnaire. The participants were asked to specify the number of times they had ingested each food item in the four days preceding the sample collection and the size of their last meal in the same period. The four-day period was chosen according to the estimated half-life of aflatoxins in mammals [30]. The information on work duties, age, and body weight was also collected.

Exposure to each specific food was calculated as a dichotomous variable (yes/no) and as a quantitative variable if consumption of that food was present in more than 50% of the questionnaires. The frequency and amount variables were transformed into a continuous variable describing the total quantity of food consumed in the preceding days.

Exposure was also classified as recent or not recent based on whether consumption was in the 48 h prior to sampling. The participants were thus classified into four groups: no consumption, consumption earlier than 48 h before sampling, between 24 h and 48 h, and in the last 24 h.

A score was calculated for each food item as the combination of quantity of the item and frequency of its consumption.

### 2.5. Data Analysis

We report proportion of positive samples and mean values of AFM1 among positive samples for consumers and non-consumers of each food item. We tested the association between urine levels of AFM1 and each food item first classified as a dichotomous variable (yes/no), then according to the time elapsed between consumption and sampling, and finally classified as a score of timing and quantity of food, when possible. We tested the equality of the median values between consumers and non-consumers in the two populations using the Wilcoxon test; the resulting *p*-values represent the probability that the two groups are two independent random samples of the same population for the distribution of AFM1 values in urine. We tested the correlation between AFM1 levels and time since exposure and the time and quantity classification of exposure using linear regression models. The analyses took into account the non-independence of samples in the same individuals. For the regression models, we substituted values below the limit of determination (< LoD) with 0; we also performed sensitivity analyses using the LoD value (0.002 ng/mL for urine), LoD/2, and LoD/ (√2).

## 3. Results

The mean age of the participating subjects was 50.2 years (range 28–67), and the mean body weight was 81.5 kg (range 60–104).

Overall, 74% of the urine samples were positive for AFM1 (LoD 0.002 ng/mL), with an average concentration of 0.042 ng/mL (range 0.002–0.399).

Urine concentration of AFM1 was not associated with consumption of any of the maize-based foods or with dairy products, either as dichotomous variables (Table 1) or when the time since consumption was taken into account or the quantity of food consumption was assessed (Table 2). The same was observed when the consumption of all maize-based foods or dairy products was pooled in a single score (Table 1 and Table 2).

Among the other cereals that can potentially be contaminated, only the consumption of “other rice-based foods” was positively associated with urine AFM1 levels. In fact, the only two people that consumed rice-based foods had 0.087 and 0.161 ng/mL of AFM1, respectively. In both cases, consumption had occurred 48 h before urine sampling, and the quantity of food was minimal. Cereal-based snack and snack cake consumption was also associated with urine AFM1 concentration; in this case, the number of subjects consuming this food was consistent, but the association was weak and compatible with random fluctuation.

Very few subjects in our sample, or even no one, consumed any of the many foods classified as at risk of contamination (Table 1). For those foods consumed by more than 5 subjects, we did not observe any positive association, with the exception of chili pepper and “other spices”; both these associations were compatible with random fluctuations, and the “other spices”, when specified, were different from each other. The association with chili pepper disappeared when we classified the exposure not simply as presence or absence, but tried to quantify it according to the time since exposure, the quantity of consumed food, and the combination of the two (Table 2). Only red meat, a food classified as not at risk, was slightly associated when time since exposure and quantity were combined (Table 2). However, the association was much weaker or absent when consumption of red meat was measured as frequency and quantity of the meat consumed (Table 1 and Table 2).

## 4. Discussion

We did not find any relevant association between urine levels of AFM1 and the consumption of maize-based or dairy products in our study population. Furthermore, the only maize-based food or dairy product that could explain a high proportion of the AFM1 positive samples (74%) is aged cheese, which is considered to be at relatively low risk of contamination. However, some subjects with very highly contaminated urine samples (>0.1 ng/mL) were not exposed to this food. In the previous analysis, we showed that occupational exposure to contaminated corn dusts and flours could not explain the vast majority of cases of AFM1 variability found in urine AFM1 levels of workers nor, obviously, in non-exposed controls [14], despite our study not being able to rule out that exposed workers may have slightly higher levels of AFM1 at the end of the work week than at the beginning.

Thus, after having analyzed any possible association between urine AFM1 levels and food or occupational exposure, we could not find any explanation for the high level of AFM1 contamination in both non-exposed controls and exposed workers.

The main limitation of the analyses presented here is that the study was designed for a different aim, i.e., to compare the AFM1 levels at the beginning and at the end of the work week in exposed workers and non-exposed controls. This design was powerful enough to study any occupational exposure, but it does not have the same validity to study food exposure. In fact, in the case of aflatoxins, contamination may occur in a single lot of a specific food, but not in many other lots of the same food. Thus, a questionnaire collecting generic information on the foods consumed in the previous four days without any analytical identification of the food origin (whether imported or not, and if so, from where) has a very low probability of capturing any association with contaminated food. This is why previous studies found inconsistent results about the association between food consumption and urine aflatoxin levels, with many surveys finding no association at all with any kinds of food [22,23,31] and others finding an association with different foods and dietary patterns [21,26,28,31,32]. In fact, the questionnaire in our study was designed to eliminate confounding in the analysis of occupational exposure due to food consumption by identifying any outliers with very high concentrations of AFM1 who had particular patterns of consumption of those foods classified as at very high risk of contamination. Nevertheless, once an unexpectedly high concentration of AFM1 in our population was observed, the food questionnaires, even with their intrinsic limitations, appeared to be a good way to check whether contaminated maize had in some way entered the food chain and arrived to humans. Moreover, when studying the possible occupational exposure, we originally decided to focus on AFM1 because of its carcinogenicity and since it is the most important urine and serum biomarker of AFB1 consumption. It is also frequently found in cow milk, cheese, and other dairy products as a result of the carryover from AFB1-contaminated foods ingested by ruminants.

Any attempt to investigate other associations between food consumption and urine AFM1 levels should take this limitation of the study design into account. In fact, in our study, two conditions were to exist to observe the association between consumption of a certain food item and urine AFM1 levels: (1) some contaminated lots are present in the food distribution in our community, and (2) there is a sufficiently high proportion of people in our study population that consumed the contaminated lots among those who declare they have consumed that food item to influence the average urine AFM1 level of the whole group. How large a proportion of consumers of the actually contaminated food we need to investigate depends on how strong the contamination is, i.e., how large the difference is between the aflatoxin level in the samples from truly exposed subjects and the background noise. For these conditions to have a high enough probability of occurring, the proportion of contaminated lots needs to be high, or the number of lots distributed in a given community needs to be low enough that the subjects who declared they had consumed that food in our study population were likely to have been exposed to the same lot. All these conditions are quite unlikely for spices, chili pepper, and “other rice products”, for which we found a slight association, because they are considered fresh for quite a long time and are produced in different places, two factors that make it very unlikely that many people in the same area have consumed the same lot. This point, together with a high *p*-value, suggests that the observed associations are due to random fluctuations, a common finding when explorative analyses are performed in adjunct to a test of hypothesis.

## 5. Conclusions

The extremely dry season of 2012 caused high contamination of corn in Northern Italy, but the relatively high urine level of aflatoxins found in a population of healthy males in the Reggio Emilia province was not associated with the consumption of foods linked to maize or cow milk or dairy products. These analyses rule out the possibility that the cause of aflatoxin in human urine is contaminated maize having entered the food chain, thus reaching humans. Other possible explanations, which are impossible to falsify with the existing data, should be considered, such as food contamination of single lots of foods not linked to a single kind of item. Furthermore, this analysis confirms that high level of contamination can be observed in populations apparently not exposed to specific sources of contamination in temperate climate countries as well.

## Figures and Tables

**Table 1:** Number of urine samples positive for aflatoxin FM1 (AFM1), means, ranges, *p*-values for AFM1 urinary levels in consumers of each kind of food compared to non-consumers.

Foods	Consumers				Non-Consumers				
	*n*	*n* (%) of Samples Positive	Mean (ng/mL)	Min–Max (ng/mL)	*n*	*n* (%) of Samples Positive	Mean (ng/mL)	Min–Max (ng/mL)	*p*-Value ^#^
**Corn flour-based products**									
Biscuits ***	3	2 (66.7)	0.014	0.013–0.014	115	85 (73.9)	0.043	0.002–0.399	-
Cakes ***	1	0 (-)	-	-	117	87 (74.4)	0.042	0.002–0.399	-
Corn cereals ***	6	3 (50.0)	0.022	0.006–0.047	112	82 (73.2)	0.043	0.002–0.399	0.214
Popcorn ***	3	1 (33.3)	0.01	0.01–0.01	115	86 (74.8)	0.043	0.002–0.399	-
Polenta (cooked or fried) ***	9	7 (77.8)	0.047	0.008–0.088	109	80 (73.4)	0.034	0.002–0.399	0.387
Other ***	4	2 (50.0)	0.025	0.007–0.043	114	85 (74.6)	0.043	0.002–0.399	-
Any corn flour-based products ***	21	13 (61.9)	0.026	0.006–0.088	97	74 (76.3)	0.045	0.002–0.399	0.299
**Fresh milk**									
Milk distributed by a coin machine *** (^)	1	0 (-)	-	-	117	87 (74.4)	0.042	0.002–0.399	-
Fresh milk from a supermarket ** (^^)	24	19 (79.2)	0.032	0.002–0.161	94	68 (72.3)	0.045	0.004–0.399	0.528
Fresh cheese **	65	53 (81.5)	0.037	0.002–0.399	53	34 (64.2)	0.050	0.003–0.180	0.606
Aged cheese **	92	64 (69.6)	0.048	0.002–0.399	26	23 (88.5)	0.025	0.003–0.110	0.553
**Fish**									
Fish *	57	46 (80.7)	0.042	0.002–0.399	61	41 (67.2)	0.0425	0.004–0.259	0.409
Shellfish *	26	20 (76.9)	0.025	0.003–0.113	92	67 (72.8)	0.0474	0.002–0.399	0.829
**Fresh fruit**									
Bananas *	59	45 (76.3)	0.032	0.002–0.161	59	42 (71.2)	0.053	0.003–0.399	0.585
Pears *	37	30 (81.1)	0.039	0.004–0.161	81	57 (70.4)	0.044	0.002–0.399	0.361
Apples *	84	62 (73.8)	0.040	0.002–0.18	34	25 (73.5)	0.048	0.003–0.399	0.619
Kiwis *	31	23 (74.2)	0.023	0.003–0.157	87	64 (73.6)	0.049	0.002–0.399	0.179
Citrus fruits *	82	59 (72.0)	0.038	0.003–0.399	36	28 (77.8)	0.052	0.002–0.259	0.096
Other *	24	18 (75.0)	0.025	0.004–0.116	94	69 (73.4)	0.047	0.002–0.399	0.352
**Meat**									
Liver (pork/bovine) ***	0	-	-	-	118	87 (73.7)	0.042	0.002–0.399	-
Beef *	97	74 (76.3)	0.044	0.003–0.399	21	13 (61.9)	0.031	0.002–0.110	0.127
Chicken *	80	55 (68.8)	0.047	0.003–0.399	38	32 (84.2)	0.034	0.002–0.259	0.771
**Cereals or cereal-based products**									
Bread **	105	75 (71.4)	0.044	0.002–0.399	13	12 (92.3)	0.032	0.003–0.157	0.504
Pasta **	112	83 (74.1)	0.042	0.002–0.399	6	4 (66.7)	0.051	0.005–0.157	0.643
Grain soups **	14	10 (71.4)	0.032	0.003–0.113	104	77 (74.0)	0.044	0.002–0.399	0.671
Muesli **	9	5 (55.6)	0.009	0.007–0.016	109	82 (75.2)	0.044	0.002–0.399	0.058
Other cereals **	61	43 (70.5)	0.034	0.003–0.161	57	44 (77.2)	0.050	0.002–0.399	0.509
Biscuits and rusks **	83	61 (73.5)	0.046	0.002–0.399	35	26 (74.3)	0.033	0.003–0.113	0.842
Snack cakes **	50	41 (82.0)	0.048	0.003–0.399	68	46 (67.6)	0.038	0.002–0.259	0.103
Cakes **	5	4 (80.0)	0.019	0.006–0.047	113	83 (73.5)	0.043	0.002–0.399	0.850
Other cereals (pieces) **	17	14 (82.4)	0.048	0.007–0.259	101	73 (72.3)	0.041	0.002–0.399	0.339
**Rice-based or rice-flour based products**									
Rice **	48	34 (70.8)	0.041	0.004–0.259	70	53 (75.7)	0.043	0.002–0.399	0.625
Rice pasta **	0	-	-	-	118	87 (73.7)	0.042	0.002–0.399	-
Other **	0	-	-	-	118	87 (73.7)	0.042	0.002–0.399	-
Biscuits **	3	1 (33.3)	0.015	0.015–0.015	115	86 (74.8)	0.043	0.002–0.399	-
Cakes **	7	2 (28.6)	0.015	0.013–0.017	111	85 (76.6)	0.043	0.002–0.399	0.027
Puffed rice **	7	5 (71.4)	0.041	0.005–0.116	111	82 (73.9)	0.042	0.002–0.399	0.950
Other **	2	2 (100.0)	0.124	0.087–0.161	116	85 (73.3)	0.040	0.002–0.399	-
**Spices**									
Pepper ***	25	18 (72.0)	0.043	0.003–0.259	93	69 (74.2)	0.042	0.002–0.399	0.325
Ginger ***	3	2 (66.7)	0.048	0.007–0.088	115	85 (73.9)	0.042	0.002–0.399	-
Nutmeg ***	1	1 (100.0)	0.008	0.008–0.008	117	45 (38.5)	0.036	0.002–0.259	-
Chili pepper ***	46	31 (67.4)	0.040	0.003–0.259	72	56 (77.8)	0.044	0.002–0.399	0.172
Other ***	4	4 (100.0)	0.083	0.004–0.259	114	83 (72.8)	0.040	0.002–0.399	-
**Nuts**									
Peanuts ***	21	17 (81.0)	0.036	0.003–0.116	97	70 (72.2)	0.044	0.002–0.399	0.420
Pistachios ***	8	5 (62.5)	0.034	0.006–0.116	110	82 (74.5)	0.043	0.002–0.399	0.585
Nuts ***	15	12 (80.0)	0.0358	0.003–0.113	103	75 (72.8)	0.0433	0.002–0.399	0.654
Almonds ***	11	8 (72.7)	0.029	0.004–0.096	107	79 (73.8)	0.044	0.002–0.399	0.463
Other ***	6	4 (66.7)	0.029	0.006–0.096	112	83 (74.1)	0.043	0.002–0.399	0.422
**Dried fruit**									
Dried figs ***	0	-	-	-	118	87 (73.7)	0.042	0.002–0.399	-
Dried prunes ***	3	2 (66.7)	0.012	0.009–0.014	115	85 (73.9)	0.043	0.002–0.399	-
Dried apricots ***	1	1 (100.0)	0.007	0.007–0.007	117	86 (73.5)	0.0427	0.002–0.399	-
Dates ***	3	1 (33.3)	0.008	0.008–0.008	115	86 (74.8)	0.043	0.002–0.399	-
Other ***	2	1 (50.0)	0.007	0.007–0.007	116	86 (74.1)	0.043	0.002–0.399	-

*** potential food source of aflatoxin; ** unlikely food source of aflatoxin; * foods not at risk for aflatoxin exposure based on the last ten years of reports from the Rapid Alert System for Food and Feed (RASFF) and the specific emergency measures for aflatoxins as reported by the qualitative analysis of the AF contamination alert reposting system and amendments (European Union Regulation 884/2014a (EU/884/2014)). *^#^* Wilcoxon test; ^ Fresh milk produced by a single farm; ^^ Milk produced by several farms.

**Table 2:** The association between urine AFM1 levels and food consumption frequency and combination of food quantity and frequency by kind of food.

Foods	Never	Time since Last Consumption (h)			Association between Urine AFM1 Levels and Food Consumption Time			Association between Urine AFM1 Levels and Combination of Food Quantity and Time		
		0–24	024–48	0≥48	Coefficient	95% CI		Coefficient	95% CI	
**Corn flour-based products**										
Corn cereals ***	112	5	1	0	−0.021	−0.037	−0.005	−0.066	−0.112	−0.021
Polenta (cooked or fried) ***	109	2	2	4	−0.006	−0.033	0.021	−0.018	−0.113	0.077
Any corn flour-based products ***	97	12	4	5	−0.020	−0.036	−0.005	−0.036	−0.067	−0.006
**Fresh milk**										
Fresh milk from a supermarket ** (^)	94	23	1	0	−0.007	−0.028	0.014	−0.022	−0.079	0.036
Fresh cheese **	53	35	18	12	0.004	−0.026	0.033	0.009	−0.074	0.093
Aged cheese **	26	66	15	11	0.008	−0.010	0.026	0.015	−0.034	0.065
**Fish**										
Fish *	61	22	21	14	−0.003	−0.024	0.018	0.011	−0.043	0.064
Shellfish *	92	8	10	8	−0.016	−0.044	0.012	−0.059	−0.130	0.013
**Fresh fruit**										
Bananas *	59	45	8	6	−0.012	−0.033	0.010	−0.046	−0.133	0.040
Pears *	81	28	7	2	−0.004	−0.024	0.015	−0.013	−0.082	0.056
Apples *	34	63	12	9	−0.015	−0.043	0.013	−0.043	−0.117	0.030
Kiwis *	87	24	2	5	−0.020	−0.039	−0.002	−0.074	−0.133	−0.015
Citrus fruits *	36	66	9	7	−0.010	−0.032	0.012	−0.026	−0.085	0.032
Other *	94	18	4	2	−0.015	−0.036	0.005	−0.037	−0.073	−0.002
**Meat**										
Beef *	21	46	33	18	0.034	0.007	0.061	0.021	−0.010	0.053
Chicken *	38	31	16	33	0.002	−0.023	0.026	−0.013	−0.070	0.044
**Cereals or cereal-based products**										
Bread **	13	93	9	3	−0.002	−0.031	0.027	0.004	−0.056	0.064
Pasta **	6	87	19	6	−0.025	−0.065	0.014	−0.005	−0.070	0.059
Grain soups **	104	3	4	7	−0.014	−0.052	0.024	−0.053	−0.174	0.067
Muesli **	109	7	1	1	−0.030	−0.043	−0.017	−0.121	−0.171	−0.070
Other cereals **	57	27	22	12	−0.016	−0.041	0.008	−0.027	−0.074	0.020
Biscuits and rusks **	35	66	9	8	0.015	−0.004	0.033	0.018	−0.024	0.059
Snack cakes **	68	34	8	8	0.021	−0.007	0.049	0.083	−0.028	0.194
Cakes **	113	3	1	1	−0.017	−0.042	0.009	−0.051	−0.106	0.005
Other cereals (pieces) **	101	11	2	4	−0.002	−0.031	0.026	0.017	−0.064	0.098
**Rice-based or rice flour-based products**										
Rice **	70	11	12	25	0.009	−0.032	0.050	−0.003	−0.093	0.087
Cakes **	111	5	0	2	−0.029	−0.043	−0.016	−0.081	−0.137	−0.026
Puffed rice cereal **	111	4	1	2	−0.009	−0.041	0.023	−0.039	−0.119	0.040
Total	62	21	12	23	−0.007	−0.038	0.025	−0.032	−0.098	0.033
**Spices**										
Pepper ***	93	16	3	6	−0.006	−0.039	0.027	−0.014	−0.109	0.080
Chili pepper ***	72	26	9	11	0.002	−0.024	0.027	0.021	−0.084	0.127
**Nuts**										
Peanuts ***	97	12	5	4	−0.005	−0.028	0.018	−0.027	−0.103	0.050
Pistachios ***	110	6	2	0	−0.008	−0.042	0.025	−0.030	−0.123	0.063
Nuts ***	103	7	4	4	−0.006	−0.034	0.023	−0.022	−0.136	0.092
Almonds ***	107	6	2	3	−0.013	−0.042	0.015	−0.054	−0.168	0.060
Other ***	112	3	0	3	−0.005	−0.038	0.029	−0.019	−0.153	0.115

*** potential food source of aflatoxin; ** unlikely food source of aflatoxin; * foods not at risk for aflatoxin exposure based on the last ten years of reports from the Rapid Alert System for Food and Feed (RASFF) and the specific emergency measures for aflatoxins as reported by the qualitative analysis of the AF contamination alert reposting system and amendments (European Union Regulation 884/2014a (EU/884/2014)); ^ Milk produced by several farms. CI—confidence interval.

## Supplementary Materials

The following are available online at https://www.mdpi.com/1660-4601/17/7/2510/s1: Table S1: Average aflatoxin levels in urine samples of the consumers and non-consumers stratified by occupational exposure.
