# Supplementary material for: Association between Urinary Levels of Aflatoxin and Consumption of Food Linked to Maize or Cow Milk or Dairy Products
Source: Int J Environ Res Public Health. 2020 Apr 6;17(7):2510. doi: 10.3390/ijerph17072510 (PMC7177871; doi:10.3390/ijerph17072510)
Supplement: Supplementary file 1 [file ijerph-17-02510-s001.zip › Table S1 Average aflatoxin levels in urine samples of the consumers and non-consumers stratified by occupational exposure.pdf]

Table S1: Average aflatoxin levels in urine samples of the consumers and non-consumers stratified by occupational exposure

Foods	Non Exposed Workers n = 30 (60 Questionnaires e 60 Urine Samples)												Exposed Workers n = 29 (58 Questionnaires e 58 Urine Samples)												Total Workers N=59 (128 Questionnaires e 128 Urine Samples)											
	Consumers						Non-Consumers						Consumers						Non-Consumers						Consumers						Non-Consumers					
		n (%) of Samples Positive						*n* (%) of Samples Positive						*n* (%) of Samples Positive						*n* (%) of Samples Positive						*n* (%) of Samples Positive						*n* (%) of Samples Positive				
	n			Mean	Min	Max	*n*			Mean	Min	Max	*n*			Mean	Min	Max	*n*			Mean	Min	Max	*n*			Mean	Min	Max	*n*			Mean	Min	Max
**Cereals or cereal-based products**																																				
**Bread** **	53	39	73.6	0.038	0.002	0.259	7	7	100.0	0.020	0.003	0.088	52	36	69.2	0.050	0.004	0.399	6	5	83.3	0.049	0.007	0.157	105	75	71.4	0.044	0.002	0.399	13	12	92.3	0.032	0.003	0.157
**Pasta** **	56	44	78.6	0.036	0.002	0.259	4	2	50.0	0.020	0.006	0.034	56	39	69.6	0.048	0.004	0.399	2	2	100.0	0.081	0.005	0.157	112	83	74.1	0.042	0.002	0.399	6	4	66.7	0.051	0.005	0.157
**Grain soup** **	11	8	72.7	0.025	0.003	0.088	49	38	77.6	0.038	0.002	0.259	3	2	66.7	0.060	0.006	0.113	55	39	70.9	0.049	0.004	0.399	14	10	71.4	0.032	0.003	0.113	104	77	74.0	0.044	0.002	0.399
**Muesli** **	6	4	66.7	0.010	0.007	0.016	54	42	77.8	0.038	0.002	0.259	3	1	33.3	0.007	0.007	0.007	55	40	72.7	0.051	0.004	0.399	9	5	55.6	0.009	0.007	0.016	109	82	75.2	0.044	0.002	0.399
**Other cereals** **	22	16	72.7	0.027	0.003	0.116	38	30	78.9	0.040	0.002	0.259	39	27	69.2	0.039	0.004	0.161	19	14	73.7	0.072	0.004	0.399	61	43	70.5	0.034	0.003	0.161	57	44	77.2	0.050	0.002	0.399
**Biscuit and rusks** **	44	34	77.3	0.039	0.002	0.259	16	12	75.0	0.027	0.003	0.096	39	27	69.2	0.056	0.005	0.399	19	14	73.7	0.039	0.004	0.113	83	61	73.5	0.046	0.002	0.399	35	26	74.3	0.033	0.003	0.113
**Snack cakes** **	18	14	77.8	0.042	0.003	0.180	42	32	76.2	0.033	0.002	0.259	32	27	84.4	0.051	0.004	0.399	26	14	53.8	0.049	0.005	0.157	50	41	82.0	0.048	0.003	0.399	68	46	67.6	0.038	0.002	0.259
**Cakes** **	4	4	100.0	0.019	0.006	0.047	56	42	75.0	0.037	0.002	0.259	1	0	0.0	-	-	-	57	41	71.9	0.050	0.004	0.399	5	4	80.0	0.019	0.006	0.047	113	83	73.5	0.043	0.002	0.399
**Other cereals (pieces)** **	10	9	90.0	0.055	0.007	0.259	50	37	74.0	0.031	0.002	0.180	7	5	71.4	0.035	0.007	0.089	51	36	70.6	0.052	0.004	0.399	17	14	82.4	0.048	0.007	0.259	101	73	72.3	0.041	0.002	0.399
**Rice-based or rice-flour based products**																																				
**Rice** **	29	21	72.4	0.052	0.004	0.259	31	25	80.6	0.022	0.002	0.096	19	13	68.4	0.024	0.005	0.083	39	28	71.8	0.062	0.004	0.399	48	34	70.8	0.041	0.004	0.259	70	53	75.7	0.043	0.002	0.399
**Rice pasta** **	0	-	-	-	-	-	60	46	76.7	0.035	0.002	0.259	0	-	-	-	-	-	58	41	70.7	0.050	0.004	0.399	0	-	-	-	-	-	118	87	73.7	0.042	0.002	0.399
**Other** **	0	-	-	-	-	-	60	46	76.7	0.035	0.002	0.259	0	-	-	-	-	-	58	41	70.7	0.050	0.004	0.399	0	-	-	-	-	-	118	87	73.7	0.042	0.002	0.399
**Biscuit** **	1	1	100.0	0.015	0.015	0.015	59	45	76.3	0.036	0.002	0.259	2	0	0.0	-	-	-	56	41	73.2	0.050	0.004	0.399	3	1	33.3	0.015	0.015	0.015	115	86	74.8	0.043	0.002	0.399
**Cakes** **	4	2	50.0	0.015	0.013	0.017	56	44	78.6	0.036	0.002	0.259	3	0	0.0	-	-	-	55	41	74.5	0.050	0.004	0.399	7	2	28.6	0.015	0.013	0.017	111	85	76.6	0.043	0.002	0.399
**Puffed rice** **	6	5	83.3	0.041	0.005	0.116	54	41	75.9	0.035	0.002	0.259	1	0	0.0	-	-	-	57	41	71.9	0.050	0.004	0.399	7	5	71.4	0.041	0.005	0.116	111	82	73.9	0.042	0.002	0.399
**Other** **	1	1	100.0	0.087	0.087	0.087	59	45	76.3	0.034	0.002	0.259	1	1	100.0	0.161	0.161	0.161	57	40	70.2	0.047	0.004	0.399	2	2	100.0	0.124	0.087	0.161	116	85	73.3	0.040	0.002	0.399
**Corn flour-based products**																																				
**Biscuit** ***	3	2	66.7	0.014	0.013	0.014	57	44	77.2	0.036	0.002	0.259	0	-	-	-	-	-	58	41	70.7	0.050	0.004	0.399	3	2	66.7	0.014	0.013	0.014	115	85	73.9	0.043	0.002	0.399
**Cakes** ***	1	0	0.0	-	-	-	59	46	78.0	0.035	0.002	0.259	0	-	-	-	-	-	58	41	70.7	0.050	0.004	0.399	1	0	0.0	-	-	-	117	87	74.4	0.042	0.002	0.399
**Corn cereal** ***	5	3	60.0	0.022	0.006	0.047	55	43	78.2	0.036	0.002	0.259	1	0	0.0	-	-	-	57	41	71.9	0.050	0.004	0.399	6	3	50.0	0.022	0.006	0.047	112	82	73.2	0.043	0.002	0.399
**Popcorn** ***	1	0	0.0	-	-	-	59	46	78.0	0.035	0.002	0.259	2	1	50.0	0.010	0.010	0.010	56	40	71.4	0.051	0.004	0.399	3	1	33.3	0.010	0.010	0.010	115	86	74.8	0.043	0.002	0.399
**Polenta (cooked or fried)** ***	8	6	75.0	0.038	0.008	0.088	52	40	76.9	0.035	0.002	0.259	1	1	100.0	0.022	0.022	0.022	57	40	70.2	0.051	0.004	0.399	9	7	77.8	0.047	0.008	0.088	109	80	73.4	0.034	0.002	0.399
**Other** ***	3	2	66.7	0.025	0.007	0.043	57	44	77.2	0.036	0.002	0.259	1	0	0.0	-	-	-	57	41	71.9	0.050	0.004	0.399	4	2	50.0	0.025	0.007	0.043	114	85	74.6	0.043	0.002	0.399
**Meat**																																				
**Liver (pork/bovine)** ***	0	-	-	-	-	-	60	46	76.7	0.035	0.002	0.259	0	-	-	-	-	-	58	41	70.7	0.050	0.004	0.399	0	-	-	-	-	-	118	87	73.7	0.042	0.002	0.399
**Beef** *	52	39	75.0	0.037	0.003	0.259	8	5	62.5	0.026	0.002	0.110	45	33	73.3	0.054	0.004	0.399	13	8	61.5	0.034	0.005	0.089	97	74	76.3	0.044	0.003	0.399	21	13	61.9	0.031	0.002	0.110
**Chicken** *	36	26	72.2	0.041	0.003	0.180	24	20	83.3	0.028	0.002	0.259	44	29	65.9	0.053	0.004	0.399	14	12	85.7	0.043	0.005	0.157	80	55	68.8	0.047	0.003	0.399	38	32	84.2	0.034	0.002	0.259
**Fish**																																				
**Fish** *	33	27	81.8	0.035	0.002	0.180	27	19	70.4	0.036	0.004	0.259	24	19	79.2	0.052	0.004	0.399	34	22	64.7	0.048	0.004	0.161	57	46	80.7	0.042	0.002	0.399	61	41	67.2	0.042	0.004	0.259
**Shellfish** *	14	11	78.6	0.023	0.003	0.087	46	35	76.1	0.039	0.002	0.259	12	9	75.0	0.028	0.004	0.113	46	32	69.6	0.056	0.004	0.399	26	20	76.9	0.025	0.003	0.113	92	67	72.8	0.047	0.002	0.399
**Fresh milk**																																				
**Milk distributed by coin machine** ***(^)	1	0	0.0	-	-	-	59	46	78.0	0.035	0.002	0.259	0	-	-	-	-	-	58	41	70.7	0.050	0.004	0.399	1	0	0.0	-	-	-	117	87	74.4	0.042	0.002	0.399
**Fresh milk from supermarket** ** (^^)	21	16	76.2	0.021	0.002	0.110	39	30	76.9	0.043	0.005	0.259	3	3	100.0	0.093	0.005	0.161	55	38	69.1	0.047	0.004	0.399	24	19	79.2	0.032	0.002	0.161	94	68	72.3	0.045	0.004	0.399
**Fresh cheese** **	31	27	87.1	0.034	0.002	0.259	29	19	65.5	0.037	0.003	0.180	34	26	76.5	0.041	0.004	0.399	24	15	62.5	0.066	0.006	0.161	65	53	81.5	0.037	0.002	0.399	53	34	64.2	0.050	0.003	0.180
**Seasoned cheese** **	46	33	71.7	0.041	0.002	0.259	14	13	92.9	0.022	0.003	0.110	46	31	67.4	0.056	0.004	0.399	12	10	83.3	0.030	0.006	0.084	92	64	69.6	0.048	0.002	0.399	26	23	88.5	0.025	0.003	0.110
**Spices**																																				
**Pepper** ***	16	13	81.3	0.043	0.003	0.259	44	33	75.0	0.033	0.002	0.116	9	5	55.6	0.043	0.004	0.113	49	36	73.5	0.051	0.004	0.399	25	18	72.0	0.043	0.003	0.259	93	69	74.2	0.042	0.002	0.399
**Ginger** ***	3	2	66.7	0.048	0.007	0.088	57	44	77.2	0.035	0.002	0.259	0	-	-	-	-	-	58	41	70.7	0.050	0.004	0.399	3	2	66.7	0.048	0.007	0.088	115	85	73.9	0.042	0.002	0.399
**Nutmeg** ***	1	1	100.0	0.008	0.008	0.008	59	45	76.3	0.036	0.002	0.259	0	-	-	-	-	-	58	41	70.7	0.050	0.004	0.399	1	1	100.0	0.008	0.008	0.008	117	45	38.5	0.036	0.002	0.259
**Chili pepper** ***	30	22	73.3	0.044	0.003	0.259	30	24	80.0	0.027	0.002	0.116	16	9	56.3	0.028	0.004	0.084	42	32	76.2	0.056	0.004	0.399	46	31	67.4	0.040	0.003	0.259	72	56	77.8	0.044	0.002	0.399
**Other** ***	1	1	100.0	0.259	0.259	0.259	59	45	76.3	0.305	0.002	0.180	3	1	33.3	0.259	0.259	0.259	55	45	81.8	0.030	0.002	0.180	4	4	100.0	0.083	0.004	0.259	114	83	72.8	0.040	0.002	0.399
**Fresh fruit**																																				
**Bananas** *	26	21	80.8	0.027	0.002	0.116	34	25	73.5	0.043	0.003	0.259	33	24	72.7	0.037	0.004	0.161	25	17	68.0	0.068	0.005	0.399	59	45	76.3	0.032	0.002	0.161	59	42	71.2	0.053	0.003	0.399
**Pears** *	21	18	85.7	0.029	0.005	0.116	39	28	71.8	0.039	0.002	0.259	16	12	75.0	0.053	0.004	0.161	42	29	69.0	0.049	0.005	0.399	37	30	81.1	0.039	0.004	0.161	81	57	70.4	0.044	0.002	0.399
**Apples** *	43	33	76.7	0.032	0.002	0.180	17	13	76.5	0.045	0.003	0.259	41	29	70.7	0.049	0.004	0.161	17	12	70.6	0.052	0.004	0.399	84	62	73.8	0.040	0.002	0.180	34	25	73.5	0.048	0.003	0.399
**Kiwi** *	17	14	82.4	0.217	0.003	0.116	43	32	74.4	0.041	0.002	0.259	14	9	64.3	0.026	0.004	0.157	44	32	72.7	0.057	0.004	0.399	31	23	74.2	0.023	0.003	0.157	87	64	73.6	0.049	0.002	0.399
**Citrus fruits** *	40	31	77.5	0.031	0.003	0.180	20	15	75.0	0.045	0.002	0.259	42	28	66.7	0.046	0.004	0.399	16	13	81.3	0.059	0.006	0.157	82	59	72.0	0.038	0.003	0.399	36	28	77.8	0.052	0.002	0.259
**Other** *	11	9	81.8	0.026	0.005	0.116	49	37	75.5	0.038	0.002	0.259	13	9	69.2	0.024	0.004	0.089	45	32	71.1	0.057	0.004	0.399	24	18	75.0	0.025	0.004	0.116	94	69	73.4	0.047	0.002	0.399
**Nuts**																																				
**Peanuts** ***	13	10	76.9	0.044	0.003	0.116	47	36	76.6	0.033	0.002	0.259	8	7	87.5	0.024	0.005	0.089	50	34	68.0	0.055	0.004	0.399	21	17	81.0	0.036	0.003	0.116	97	70	72.2	0.044	0.002	0.399
**Pistachios** ***	7	4	57.1	0.038	0.006	0.116	53	42	79.2	0.035	0.002	0.259	1	1	100.0	0.022	0.022	0.022	57	40	70.2	0.051	0.004	0.399	8	5	62.5	0.034	0.006	0.116	110	82	74.5	0.043	0.002	0.399
**Nuts** ***	11	8	72.7	0.025	0.003	0.096	49	38	77.6	0.038	0.002	0.259	4	4	100.0	0.057	0.010	0.113	54	37	68.5	0.049	0.004	0.399	15	12	80.0	0.036	0.003	0.113	103	75	72.8	0.043	0.002	0.399
**Almonds** ***	7	6	85.7	0.024	0.004	0.096	53	40	75.5	0.037	0.002	0.259	4	2	50.0	0.047	0.004	0.089	54	39	72.2	0.050	0.004	0.399	11	8	72.7	0.029	0.004	0.096	107	79	73.8	0.044	0.002	0.399
**Other** ***	5	3	60.0	0.037	0.006	0.096	55	43	78.2	0.035	0.002	0.259	1	1	100.0	0.007	0.007	0.007	57	40	70.2	0.051	0.004	0.399	6	4	66.7	0.029	0.006	0.096	112	83	74.1	0.043	0.002	0.399
**Dried fruit**																																				
**Dried figs** ***	0	-	-	-	-	-	60	46	76.7	0.035	0.002	0.259	0	-	-	-	-	-	58	41	70.7	0.050	0.004	0.399	0	-	-	-	-	-	118	87	73.7	0.042	0.002	0.399
**Dried prunes** ***	1	1	100.0	0.014	0.014	0.014	59	45	76.3	0.036	0.002	0.026	2	1	50.0	0.009	0.009	0.009	56	40	71.4	0.051	0.004	0.399	3	2	66.7	0.012	0.009	0.014	115	85	73.9	0.043	0.002	0.399
**Dried apricots** ***	0	-	-	-	-	-	60	46	76.7	0.035	0.002	0.259	1	1	100.0	0.007	0.007	0.007	57	40	70.2	0.051	0.004	0.399	1	1	100.0	0.007	0.007	0.007	117	86	73.5	0.043	0.002	0.399
**Dates** ***	2	1	50.0	0.008	0.008	0.008	58	45	77.6	0.036	0.002	0.259	1	0	0.0	-	-	-	57	41	71.9	0.050	0.004	0.399	3	1	33.3	0.008	0.008	0.008	115	86	74.8	0.043	0.002	0.399
**Other** ***	1	0	0.0	-	-	-	59	46	78.0	0.035	0.002	0.259	1	1	100.0	0.007	0.007	0.007	57	40	70.2	0.051	0.004	0.399	2	1	50.0	0.007	0.007	0.007	116	86	74.1	0.043	0.002	0.399

*** potential food source of aflatoxin; ** unlikely food source of aflatoxin; * foods not at risk for aflatoxin exposure. based on the last ten years of reports from the Rapid Alert System for Food and Feed (RASFF) and the specific emergency measures for aflatoxins as reported in the European Union Regulation 884/2014a qualitative analysis of the AF contamination alert reposting system (EU/884/2014) and amendments;

^ Fresh milk produced by a single farm; ^^ Milk produced by several farms
